# Integrated Bioinformatic Analysis Identifies Networks and Promising Biomarkers for Hepatitis B Virus-Related Hepatocellular Carcinoma

**DOI:** 10.1155/2020/2061024

**Published:** 2020-07-28

**Authors:** Yun Ji, Yue Yin, Weizhen Zhang

**Affiliations:** Department of Physiology and Pathophysiology, Peking University Health Science Center, Beijing 100191, China

## Abstract

Chronic infection with hepatitis B virus (HBV) has long been recognized as a dominant hazard factor for hepatocellular carcinoma (HCC) and accounts for at least half of HCC instances globally. However, the underlying molecular mechanism of HBV-linked HCC has not been completely elucidated. Here, three microarray datasets, totally containing 170 tumoral samples and 181 adjacent normal tissues from the liver of patients suffering from HBV-related HCC assembled from the Gene Expression Omnibus (GEO) database, were subjected to integrated analysis of differentially expressed genes (DEGs). Subsequently, the analysis of function and pathway enrichment as well as the protein-protein interaction network (PPI) was performed. The ten hub genes screened out from the PPI network were further subjected to expression profile and survival analysis. Overall, 329 DEGs (67 upregulated and 262 downregulated) were identified. Ten DEGs with the highest degree of connectivity included cyclin-dependent kinase 1 (CDK1), cyclin B1 (CCNB1), cyclin B2 (CCNB2), PDZ-binding kinase (PBK), abnormal spindle microtubule assembly (ASPM), nuclear division cycle 80 (NDC80), aurora kinase A (AURKA), targeting protein for xenopus kinesin-like protein 2 (TPX2), kinesin family member 2C (KIF2C), and centromere protein F (CENPF). Kaplan-Meier analysis unveiled that overexpression levels of KIF2C and TPX2 were relevant to both the poor overall survival and relapse-free survival. In summary, the hub genes validated in the present study may provide promising targets for the diagnosis, prognosis, and therapy of HBV-associated HCC. Additionally, our work uncovers various crucial biological components (e.g., extracellular exosome) and signaling pathways that participate in the progression of HCC induced by HBV, serving comprehensive knowledge of the mechanisms regarding HBV-related HCC.

## 1. Introduction

Based on the new statistics in 2018, hepatocellular carcinoma (HCC) has been estimated to be the seventh most prevalent cancer and the third major cause of tumor-related death worldwide [1]. One of the leading risk factors for HCC is hepatitis B virus (HBV). HBV is the primary reason for HCC in Africa and East Asia, accounting for approximately 80 percent of all cases of HCC associated with the virus worldwide [2]. As an oncogenic virus, HBV has been known as a trigger of HCC even in the absence of cirrhosis. However, the risk of HCC is affected by heredity, infection, and nutritional and lifestyle factors in those infected with HBV [3]. The mechanisms of HBV-related HCC have been proposed to be linked with chronic inflammation and hepatocellular regeneration [4]. More importantly, the integration of HBV DNA into the host genome activates the host genes responsible for cell survival, proliferation, and immortalization [5]. In addition, the epigenetic regulation of tumor suppressor genes by HBV protein is involved in the initiation and progression of HCC induced by HBV [6]. While many attempts have been made to comprehend the mechanism for HBV-triggered HCC, the prevention and treatment of the disease remain a significant challenge. Effective biomarkers for the diagnosis, prognosis, and therapy of HBV-related HCC are therefore urgently required in order to enhance the survival rate of patients.

High-throughput sequencing (RNA-seq) and microarrays have been commonly used in the molecular diagnosis and discovery of novel cancer biomarkers [7]. These techniques are excellent options to profile massive gene expression datasets so that the mechanisms underlying HCC are interpreted in depth. RNA-seq and microarrays have so far been used as evidence for hundreds of differentially expressed genes enriched by various signaling pathways and biological processes to reveal molecular markers clinically for a specific tumor type [8]. Studies related to comparative DEG analysis between HBV-induced tumor and normal tissues in the liver are limited. The potential molecular mechanisms of HBV-related tumor in the liver may therefore be clarified through the identification of hub genes (the genes that possess plenty of interactions with other genes and typically play vital roles in the regulation of signaling pathways and biological processes [9]) using bioinformatic analysis, thus conducing to developing efficient novel diagnostic and therapy strategies.

Since the variability occurs in different projects, bioinformatic techniques can assist in acquiring more accurate biomarkers by integrating information from multiple projects. In the present study, we integrated three datasets obtained from the GEO database and attempted to identify hub genes and pathways and to screen for the potential therapeutic targets of HCC induced by HBV infection using bioinformatic analysis.

## 2. Materials and Methods

### 2.1. Data Collection

**Figure 1:**
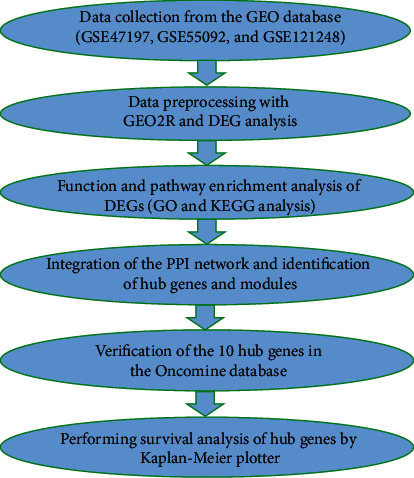
The workflow diagram of data acquisition, preprocessing, analysis, and validation.

The workflow of this study is presented in Figure 1. The dataset search was performed by using terms (“HCC” [Description] OR “tumor” [Description] OR “hepatocellular carcinoma” [Description]) AND (“HBV” [Description] OR “hepatitis B virus” [Description])) AND “Homo sapiens” [Organism]) AND “Expression profiling by array” [DataSet Type] in the Gene Expression Omnibus (GEO) database (https://www.ncbi.nlm.nih.gov/geo/) founded by the National Center for Biotechnology Information. The datasets were required to fulfill the following criteria: (1) the data were obtained from a clinical study, not the study focusing on a cell line or specific immune cells; (2) the sample size of the dataset had to be greater than one hundred; and (3) the comparison subjects had to be HBV-related tumoral vs. adjacent nontumoral liver tissues. Three microarray datasets represented different racial populations (France, United States, and Singapore) including GSE47197, GSE55092, and GSE121248 which were finally selected from the search results. Data from GSE47197 was based on GPL16699 platforms (Agilent-039494 SurePrint G3 Human GE v2 8x60K Microarray 039381) and contained 63 nontumoral and 61 tumoral samples of the liver infected with HBV (last update date: 23 April 2018). Gene expression profiling from GSE55092 and GSE121248 were based on GPL570 platforms ([HG-U133_Plus_2] Affymetrix Human Genome U133 Plus 2.0 Array). The GSE55092 dataset (last update date: 25 March 2019) included 81 and 39 samples, respectively, from nontumor and tumor areas of liver tissues from HBV-associated HCC patients, while the GSE121248 dataset (last update date: 25 March 2019) contained 37 adjacent normal tissues and 70 tumoral samples of liver tissues from patients suffering from HCC induced by HBV infection.

### 2.2. Data Preprocessing and DEG Analysis

GEO2R (https://www.ncbi.nlm.nih.gov/geo/geo2r/), an interactive internet instrument provided by the National Center for Biotechnical Information, was used 5for identifying DEGs between nontumoral and tumoral liver samples from HCC patients with HBV infection. The principle of GEO2R is based on using the GEOquery and limma R packages from the Bioconductor project which carries out a comparison on processed data tables of microarray data provided by an initial submitter [10–12]. As a cutoff criterion, adjusted *P* value < 0.05 and ∣logFC | ≥1.0 were used for identifying aberrantly expressed genes which were visualized by a volcano plot. The final dataset of DEGs was achieved through the overlapping of the three datasets using an online tool that generates a textual and graphical output (http://bioinformatics.psb.ugent.be/webtools/Venn/).

### 2.3. Function and Pathway Enrichment Analysis of DEGs

The enrichment analysis of DEG-associated functions and pathways was evaluated by the Gene Ontology (GO) and Kyoto Encyclopedia of Genes and Genomes (KEGG) databases. The GO project provides a useful approach in which biological functions are interpreted and displayed, and it covers three concepts including Biological Process (BP), Cellular Component (CC), and Molecular Function (MF). The KEGG pathway represents the integration of biological molecular interaction and reaction networks, curated from academic literatures. GO and KEGG analyses were performed with the Database for Annotation, Visualization and Integrated Discovery (DAVID, version 6.8) (https://david.ncifcrf.gov/) [13] and ClueGO (version 2.5.4) [14], an app operated in Cytoscape (version 3.7.1, https://cytoscape.org/) [15]. A *P* value < 0.01 and gene counts ≥ 8 (for KEGG analysis: ≥6) were considered statistically significant (the gene count is an arbitrary value that can be set to as low as 3 under the premise of preserving important terms judged by a user). To connect the terms in the network, ClueGO utilizes kappa statistics in which the kappa score was set as ≥0.4 in the current study.

### 2.4. Integration of the PPI Network and Identification of Hub Genes and Modules

The protein-protein interaction (PPI) network was constructed by submitting a DEG list to the STRING (Search Tool for the Retrieval of Interacting Genes/Proteins) database (http://string-db.org/, version 11.0) [16]. The minimum required interaction score was 0.7 (high confidence). Posteriorly, the PPI network was displayed with Cytoscape software (version 3.7.1). By calculating the connectivity degree (the number of other nodes that interact directly with one specific node) of each protein node using CytoHubba (a plugin in Cytoscape) [17], the top ten genes with the highest connectivity degrees were recognized as hub genes for HBV-associated HCC. The modules in the PPI network were evaluated with an MCODE plugin of Cytoscape software [18] by using the default parameter, followed by a pathway analysis with Reactome [19] (https://reactome.org/) Pathway Browser version 3.6.

### 2.5. Verification of the Hub Genes in the Oncomine Database

The ten candidate hub genes were validated by Oncomine (https://www.oncomine.org/) which is a microarray cancer database with a web-based data mining platform to support the analysis of genome-wide expression [20]. Datasets were filtered by cancer type (liver cancer) and analysis type (cancer vs. normal analysis) and then were set a threshold by a *P* value less than 1 × 10^−4^ and genes ranked top 10%. According to the sample size, patient type, and overexpression/copy number gain gene rank, results from Roessler et al. [21] and Guichard et al. [22] displayed in Oncomine were selected for the evaluation of the hub gene expression levels in liver tissues from HBV-related HCC patients, compared with those in normal liver tissues. Oncomine offers statistical significance by using Student's *t*-test. The thresholds were set to “1*E* − 4, 2, and 10%,” respectively, for the *P* value, fold change, and gene rank.

### 2.6. Survival Analysis of Hub Genes

Survival analysis was conducted in a Kaplan-Meier plotter online tool (http://kmplot.com/analysis/) [23, 24]. As highlighted by Goel et al. [25], the Kaplan-Meier prediction is one of the best methods for calculating the proportion of individuals who live after treatment for a period of time. This method previously has been widely used in the survival analysis for multiple cancer types including hepatocellular carcinoma [26, 27], breast cancer [28], ovarian cancer [29], pancreatic carcinoma [30], and gastric cancer [31]. Based on multiple databases handled by the integration of gene expression and clinical data, a Kaplan-Meier survival plotter enables survival prediction over time, even when patients quit or are observed for different durations [25]. It provides the comparison of the survival rate between patient cohorts with low and high expression level of a particular gene and calculates the logrank *P* value and hazard ratio (HR) with 95% confidence intervals. In hepatitis virus-infected liver cancer patients, the Kaplan-Meier plotter mRNA liver cancer database [24] was implemented to estimate prognostic values for hub genes. Patients were split by autoselect best cutoff (all potential cutoff values are assessed from the lower to upper of the quartiles, and the most effective threshold is used as a cutoff). Hepatitis virus as a risk factor was included in the analysis. Meanwhile, an alcohol consumption factor was eliminated.

### 2.7. Statistical Analysis

The adjusted *P* values of data from the GEO DataSets were calculated with GEO2R software. Data with an adjusted *P* value less than 0.05 was regarded as statistically significant. The GO terms and the KEGG pathways were considered significantly enriched with a value of *P* < 0.01. For the Kaplan-Meier analysis, hazard ratios (HR) with 95% confidence intervals and logrank *P* values were calculated. A logrank *P* value less than 0.05 was taken as a statistically significant difference.

## 3. Results

### 3.1. Identification of DEGs in HBV-Associated HCC

**Figure 2:**
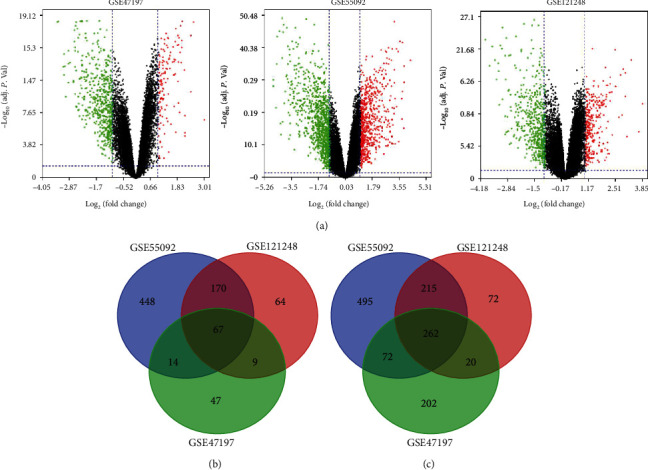
Volcano plots and Venn diagrams of differentially expressed genes (DEGs) selected from three Gene Expression Omnibus (GEO) datasets. (a) Volcano plots of DEGs in normal and tumoral liver samples of HCC patients with HBV infection in GSE47197, GSE55092, and GSE121248. DEGs were filtered by adjusted *P* value < 0.05 and ∣log_2_ (fold change) | ≥1. The red and green dots display the distribution of all the significant upregulated (red dots) and downregulated (green dots) DEGs in the three datasets, respectively. (b, c) The Venn diagrams of overlapping DEGs from an intersection of upregulated and downregulated genes in the three datasets.

Gene expression profiles of HBV-induced tumor and adjacent normal liver tissue were obtained from GSE47197, GSE55092, and GSE121248. In total, the three datasets contained 181 nontumoral and 170 tumoral samples of the liver infected with HBV. Following the cutoff criterion (adjusted *P* value < 0.05 and ∣logFC | ≥1.0), 709 DEGs (137 upregulated and 572 downregulated DEGs) were attained from GSE47197, 1743 DEGs including 699 upregulated and 1044 downregulated DEGs were identified from GSE55092, and 879 DEGs (310 upregulated and 569 downregulated DEGs) were extracted from GSE121248. The distribution of DEGs was illustrated by volcano plots as shown in Figure 2(a). The Venn diagrams showed the number of overlapping genes across the three datasets. A total of 329 genes overlapping with 67 upregulated and 262 downregulated DEGs were found within the common region (Figure 2(b) and Table 1).

### 3.2. Gene Ontology Enrichment Analysis of DEGs in HBV-Related HCC

**Figure 3:**
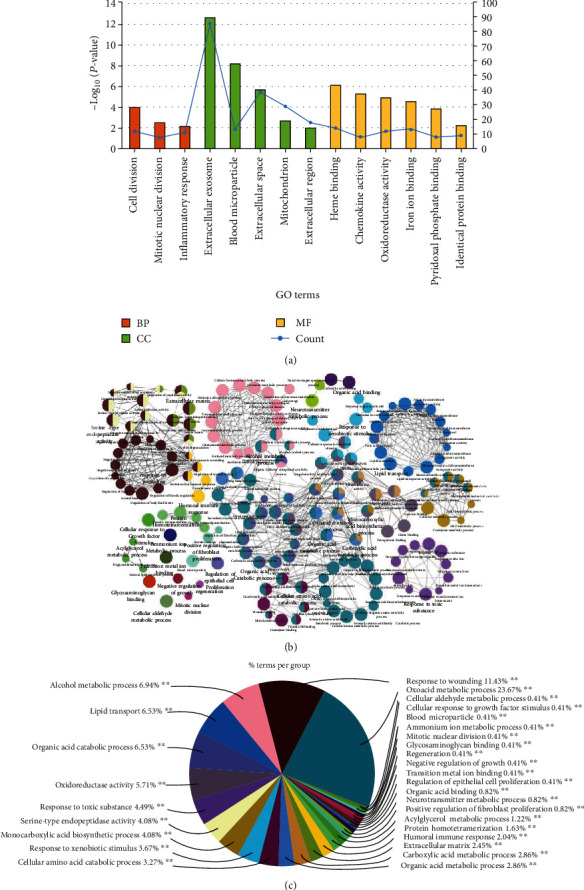
Gene Ontology (GO) enrichment analysis of DEGs following the evaluation by (a) the DAVID functional annotation and (b, c) ClueGO plugin. (a) The histogram with orange, green, and yellow shows GO terms Biological Process (BP), Cellular Component (CC), and Molecular Function (MF), respectively. (b) Functional connection of the enriched categories for DEGs (kappa score ≥ 0.4). The GO terms are depicted as nodes, whose size represents the degree of significance. (c) The pie chart shows the clusters of GO terms that were labeled with diverse colors.

To gain a more profound understanding of the function of DEGs, GO function analysis was carried out using both DAVID and ClueGO. According to the results from DAVID (Figure 3(a) and Table S1), the GO annotation regarding biological processes significantly regulated by the DEGs included “cell division,” “mitotic nuclear division,” and “inflammatory response” (*P* < 0.01). The category from cellular components was associated with “extracellular exosome,” “blood microparticle,” “extracellular space,” “mitochondrion,” and “extracellular region” (*P* < 0.01). The ontology source from molecular functions of DEGs was involved in “heme binding,” “chemokine activity,” “oxidoreductase activity,” “iron ion binding,” “pyridoxal phosphate binding,” and “identical protein binding” (*P* < 0.01). Based on the analysis of ClueGO, the resulting terms and their network connections are shown in Figure 3(b) (detailed information in Supplementary PDF file 1), and the percentage of core terms for each group is displayed in Figure 3(c).

### 3.3. KEGG Pathway Enrichment Analysis in HBV-Induced HCC

**Figure 4:**
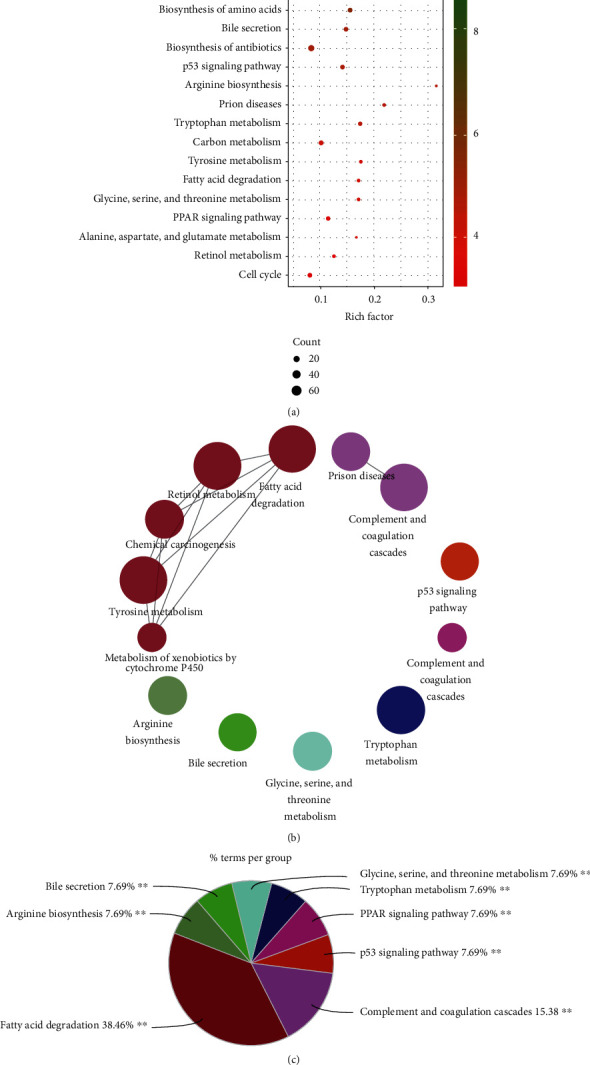
Kyoto Encyclopedia of Genes and Genomes (KEGG) enrichment analysis of DEGs based on the DAVID functional annotation and ClueGO plugin of Cytoscape. (a) KEGG enrichment analyzed by DAVID is displayed by a scatter plot. (b) The network with terms connected to its kappa score point (≥0.4) as evaluated by the ClueGO plugin. (c) Overview of the chart containing the most significant KEGG terms with the corresponding colors.

Following the functional annotation analysis by DAVID, DEGs were significantly enriched in “metabolic pathways,” “complement and coagulation cascades,” “biosynthesis of amino acids,” “bile secretion,” “biosynthesis of antibiotics,” “p53 signaling pathway,” “carbon metabolism,” “cell cycle,” etc. for the KEGG pathway (Figure 4(a) and Table S2), while results from ClueGO indicated that pathways including “fatty acid degradation,” “complement and coagulation cascades,” “p53 signaling pathway,” “arginine biosynthesis,” “bile secretion,” “glycine, serine, and threonine metabolism,” “tryptophan metabolism,” and “PPAR signaling pathway” were significantly enriched (Figures 4(b) and 4(c)).

### 3.4. Screening of Hub Genes and Module Analysis from DEGs in the Protein-Protein Interaction (PPI) Network

**Figure 5:**
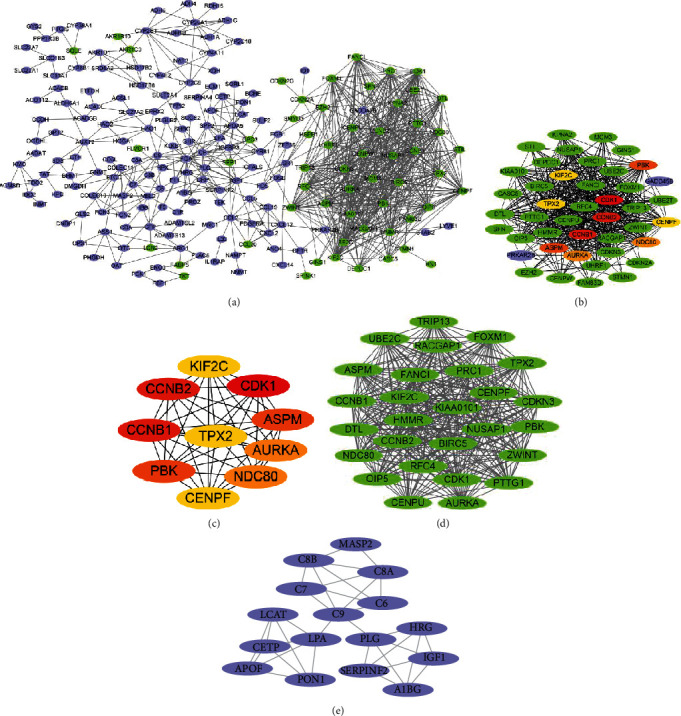
Protein-protein interaction (PPI) network, hub gene screening, and module analysis. (a) The PPI network constructed using STRING 11.0 was visualized by Cytoscape. The upregulated genes are shown in green, while the downregulated genes are shown in bluish violet. (b) The network directly associated with the top ten hub genes identified by CytoHubba. (c) The network of the top ten hub genes filtered according to the degree method provided by CytoHubba. (d, e) The key modules identified using the MCODE plugin of Cytoscape, which contains 27 nodes/344 edges and 16 nodes/34 edges, respectively.

The PPI network with 325 nodes and 843 edges predicted using STRING 11.0 with a score ≥ 0.7 (high confidence) was visualized by Cytoscape as shown in Figure 5(a) (detailed information in Supplementary PDF file 2). The top ten hub genes in the PPI network were identified by the connectivity degree (Table 2). The top ten nodes with the highest degrees included cyclin-dependent kinase 1 (CDK1, degree = 41), cyclin B1 (CCNB1, degree = 37), cyclin B2 (CCNB2, degree = 37), PDZ-binding kinase (PBK, degree = 34), abnormal spindle microtubule assembly (ASPM, degree = 34), nuclear division cycle 80 (NDC80, degree = 33), aurora kinase A (AURKA, degree = 33), targeting protein for xenopus kinesin-like protein 2 (TPX2, degree = 32), kinesin family member 2C (KIF2C, degree = 32), and centromere protein F (CENPF, degree = 32). The network of genes most closely related to the 10 hub genes is shown in Figure 5(b). Meanwhile, the linkage between the 10 core genes is presented in Figure 5(c). Additionally, we picked out two modules with the highest degree from the PPI network by using MCODE as shown in Figures 5(d) and 5(e). Following the pathway analysis by Reactome, the DEGs in module 1 were primarily enriched in “cell cycle, mitotic,” “cell cycle,” and “cell cycle checkpoints,” while those in module 2 were mainly gathered in “complement cascade” and “innate immune system”.

### 3.5. Expression Level Validation and Kaplan-Meier Plot of Hub Genes

**Figure 6:**
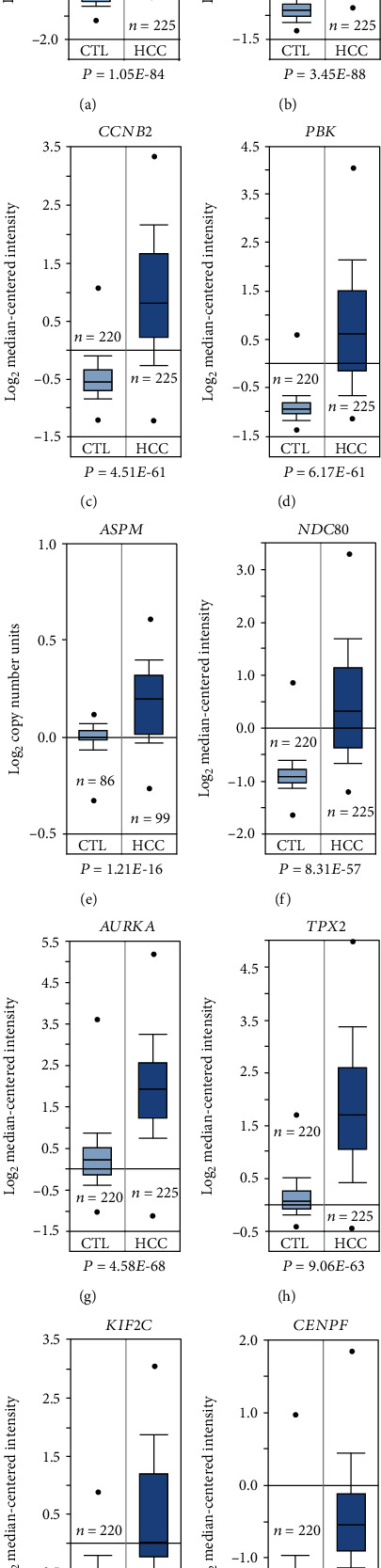
Validation of the expression level of the ten hub genes between the clinical liver samples from HBV-related HCC patients and those from healthy individuals. All the relative expression levels of hub genes were based on the dataset from Roessler et al., except for that of ASPM (Guichard et al.).

**Figure 7:**
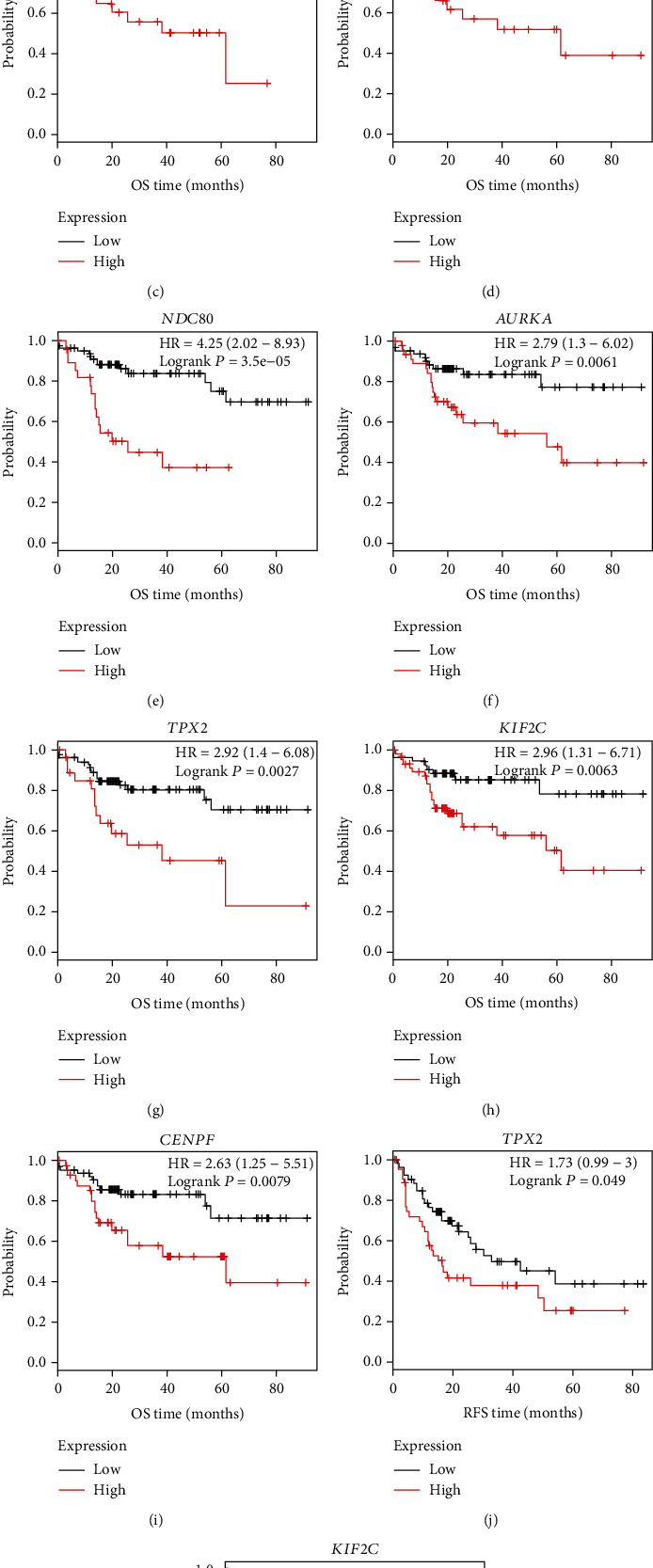
Kaplan-Meier plotter reveals the overall survival (OS) and relapse-free survival (RFS) curves with a significant difference concerning the hub genes in a liver cancer RNA-seq cohort. As risk factors, alcohol consumption was excluded and hepatitis virus was included in the analysis. The analysis was run on 111 and 103 patients for OS and RFS, respectively. *P* < 0.05 was considered a statistically significant difference.

In order to confirm the validity of the differential expressions for transcriptional level, the hub genes were validated using the Oncomine database. All the ten hub genes (CDK1, CCNB1, CCNB2, PBK, ASPM, NDC80, AURKA, TPX2, KIF2C, and CENPF) were verified to be significantly upregulated in tumor tissues from the liver of HBV-HCC patients, compared with the normal liver tissues (*P* < 0.01) (Figures 6(a)–6(j)). The survival curves of the ten hub genes were visualized by Kaplan-Meier plots. Hepatitis virus-related HCC patients with high levels of CDK1, CCNB1, PBK, ASPM, NDC80, AURKA, TPX2, KIF2C, and CENPF present a shorter overall survival (*P* < 0.05) which represents the time from randomization to death (Figures 7(a)–7(i)). These genes are valuable in the diagnosis in a clinical study of HBV-HCC patients. The unfavorable prognostics of relapse-free survival (RFS, defined as the probability of survival time until the first of relapse or death) in the patients was, however, only observed in high expression of KIF2C and TPX2 (*P* < 0.05) (Figures 7(j) and 7(k)). Therefore, KIF2C and TPX2 may be considered biomarkers for the prognosis of patients suffering from HBV-associated HCC.

## 4. Discussion

Since HBV infection continues to be a risk to public health, HBV-related HCC will still be one of the major cancers worldwide with serious economic impact on healthcare systems [32]. To develop effective diagnosis and treatment strategies, it is crucial to understand the molecular mechanisms underlying HCC induced by HBV infection. In the present study, three distinct group profile datasets were incorporated to identify the DEGs by using bioinformatic techniques. We identified a total of 67 upregulated and 262 downregulated DEGs, which are clustered based on the functions and signaling pathways by enrichment analysis. Following the results from GO enrichment analysis, most of the DEGs function in “cell division,” “mitotic nuclear division,” and “inflammatory response.” These functional categories have been closely associated with the development and progression of cancer [33–35]. Then again, the enriched KEGG pathways involved primarily in “metabolic pathways,” “complement and coagulation cascades,” “biosynthesis of amino acids,” “bile secretion,” “biosynthesis of antibiotics,” “p53 signaling pathway,” “carbon metabolism,” and “cell cycle” are all connected with tumor formation and progression [36–43]. Thus, the screened DEGs may be valuable in the diagnosis and prognosis of HBV-related HCC. In order to investigate the interplay of the DEGs, a network of PPI was established and 10 hub genes upregulated in tumor tissues of HBV-associated HCC including CDK1, CCNB1, CCNB2, PBK, ASPM, NDC80, AURKA, TPX2, KIF2C, and CENPF were screened out. According to the results from the Kaplan-Meier plotter, CDK1, CCNB1, PBK, ASPM, NDC80, AURKA, TPX2, KIF2C, and CENPF were associated with the unfavorable prognosis for patients with HCC induced by virus, and only high KIF2C and TPX2 expression, however, was linked with worse RFS. Recently, reports from in silico analysis have also identified DEGs for HBV-related HCC. Chen et al. [44], for example, have analyzed the GSE32323 gene expression profile containing 21 HBV-positive samples, of which 11 are liver cirrhosis and 10 are HCC tissues. Four of the 10 hub DEGs identified including CDK1, CCNB1, CCNB2, and KIF2C are in accordance with our results. However, results from Chen et al. were based on a dataset with small sample size, and the comparisons were made on HBV-related tumor tissue with cirrhosis samples, not normal tissues. Another published work was performed by using four microarray datasets totally containing 542 samples for bioinformatic analysis of DEGs between HCC and normal samples [45]. Three of their identified DEGs (CCNB1, CCNB2, and TPX2) are consistent with our findings. Nonetheless, it should be pointed out that the majority of their collected samples (460 of the 542 samples) were based on GSE14520 which was not designated as HBV-infected samples. By contrast, we analyzed three datasets with 351 samples, each of which consisted of over 100 samples from nontumoral and tumoral samples of liver tissues infected with HBV. The hub genes identified in the present study may therefore be more reliable.

Among the 329 DEGs, 86 are involved in “extracellular exosome” according to our enrichment analysis result. Exosome is defined as a nanosized vesicle enriched with bioactive molecules (nucleic acids, lipids, proteins, and metabolites), which is secreted by almost all types of cells including normal and tumor cells [46]. Exosomes are capable of transmitting signals between cells and triggering activation or suppression of multiple signaling pathways in recipient cells [47]. It has been known that tumor-derived exosomes are closely related to oncogenesis and tumor cell migration [48]. A recent study from Chen et al. [49] demonstrated that HCC-derived exosomes induce a progression and recurrence of HCC by epithelial-mesenchymal transition, which is associated with the activation of mitogen-activated protein kinase (MAPK)/extracellular signal-regulated kinase (ERK) signaling. Hence, the exosomes that derive from HCC may serve as promising biomarkers for the diagnosis and therapy of HCC, through delivering a range of bioactive molecules such as RNAs and proteins. Other notable GO terms of interest enriched by DEGs include “iron ion binding” and “heme binding.” Cancer cells have been well known to be addicted to iron, which is modulated by aberrant iron metabolic proteins. Iron depletion results in dramatically changes in cancer cells including global histone and DNA methylation [50]. Apart from inducing a progression to tumor in the presence of cirrhosis, an overload of iron in hepatic tissue may directly lead to HCC [51]. In contrast, a depletion of iron has been found to suppress the growth of HCC in an experimental study [52]. Targeting iron binding and its associated pathway has been proposed as novel cancer therapy [53]. Heme binding protein-released heme exhibits highly prooxidative and proinflammatory effects [54] and thereby may accelerate the development and progression of HCC. The medication such as biguanides targeting heme binding has recently been reported to display an antineoplastic effect [55]. It should be noted that KEGG enrichment analysis in the present study reveals various amino acid-associated metabolism pathways linked with HBV-related HCC including “biosynthesis of amino acids,” “arginine biosynthesis,” “tryptophan metabolism,” “tyrosine metabolism,” “glycine, serine, and threonine metabolism,” and “alanine, aspartate, and glutamate metabolism.” Results from metabolomics unveil that serum levels of serine, glutamate, phenylalanine, ornithine, and tyrosine are remarkably elevated in both patients suffering from HBV infection and HCC, compared with healthy subjects [56]. The aberrant metabolism of amino acids in tumor tissues may deliver novel diagnostic and therapeutic opportunities for HCC. For example, arginine depletion by targeting arginase has been regarded as a strategy to mitigate HCC, due to the arginine auxotrophy of certain tumor cells with low capacity of arginine synthesis [57]. Consistently, all the six DEGs (GLS2, ASS1, FOLH1B, AGXT2, CPS1, and GPT2) enriched in “arginine biosynthesis” in our results were significantly downregulated in tumor hepatic tissue from HBV-infected patients compared with adjacent nontumor tissues. Hence, the regulation of key metabolic enzymes of amino acids may conduce to intervening with the development of HCC.

CDK1, a master kinase regulating the mammalian cell cycle, was found to be one of the hub genes with the highest degree of connectivity. The target genes of CDK1 previously have been identified which are involved in DNA replication, chromosome segregation, transcriptional activity, cell morphogenesis, and genome stability [58]. Furthermore, recent progress reported by Ravindran Menon et al. has unveiled that CDK1 expedites tumor initiation through the interaction with Sox2 (sex determining region Y-box 2) [59]. It has been demonstrated that CDK1 is highly expressed in HCC and facilitates tumor progression by means of CDK1-PDK1-*β*-catenin signaling [60]. Application of an inhibitor targeting CDK1 (e.g., RO3306, BA-12, and BP-14) provides antitumor responses in HCC via suppressing the cell proliferation and viability [60–62]. However, cyclin B1-CDK1 kinase was reported to be sustainedly activated by HBV X (HBx) protein, leading to an inhibition in the growth of HCC cells [63]. Other essential regulators related to the cell cycle screened in our study include CCNB1 and CCNB2 encoding B-type cyclin protein (cyclin B). Cyclin B1 serves to initiate the transition of the G2 to M phase by binding with CDK1 and provide checkpoint in the G2/M phase via the cyclin B1-CDC2 complex. Results from human colorectal cancer have shown that overexpression of CCNB1 induced by Chk1 accelerates cancer cell proliferation and tumor growth [64]. In parallel with our study, CCNB1 has been shown as a prognostic indicator for HCC [65]. Recent in vitro evidence reveals an indispensable role of CCNB1 in the proliferation of human hepatocellular carcinoma cells, which is driven by forkhead box protein M1 (FOXM1) [65]. Importantly, cyclin B1 was verified to be highly associated with the recurrence of HBV-related HCC, thus being a candidate biomarker for HBV-HCC patients after surgery [66]. Similar to CCNB1, an aberrant expression of CCNB2 may lead to impaired G2/M checkpoint, followed by DNA damage and mutations and even oncogenesis. CCNB2 has recently been involved in cell proliferation and migration through CCNB2/PLK1 in a hepatocarcinoma cell line [67]. In contrast, downregulation of CCNB2 by silencing the upstream regulator karyopherin subunit-*α* 2 (KPNA2) induces cell cycle arrest in the G2/M phase and stagnant cell proliferation in HCC cells [68]. Recent evidence reveals that DLEU2 (Deleted In Lymphocytic Leukemia 2) and HBx cooperate to activate the transcription of CCNB2 in HBV-replicating HCC cells [69], offering a confirmation in the role of CCNB2 in HBV-HCC. The oncogenic function of CENPF, a member of kinetochore proteins, in HCC has also been proven to be associated with mitotic progression (G2/M transition) [70]. This gene has been considered a biomarker for the early diagnosis of HCC [71]. PBK previously was identified as a mitogen-activated protein kinase targeting malignancy [72]. In terms of mechanism, the role of PBK in tumorigenesis has been reported to be linked with the FoxM1/PBK/*β*-catenin axis [73]. More recently, a study from Yang et al. reveals that an overexpression of PBK facilitates the migration and invasion of HCC cells by activating the ETV4-uPAR pathway [74]. ASPM has been known as one of the key factors in oncogenesis through the regulation of cell proliferation and cell cycle progression [75]. In addition, ASPM acts as a molecular marker for predicting enhanced invasive/metastatic potential of HCC [76]. Although the regulatory mechanism of ASPM in HBV-HCC has not been reported, ASPM was demonstrated to be modulated by viral nonstructural protein 5A (NS5A) in HCV-HCC [77]. NDC80 also plays a key role in accelerating the development of HCC. As summarized in detail by Ju et al. [78], NDC80 elicits HCC progression via accelerating cell proliferation and the formation of tumor colony and inducing cell cycle arrest at the S phase. In line with our study, experimental evidence indicated a high expression of NDC80 in HBV-related HCC tissues [79]. Knockdown of NDC80 expression using shRNA inhibited the proliferation of hepatoma cells transcribed with the HBV genome [79]. Aurora A kinase (AURKA) is a member of Ser/Thr family kinase involved in the regulation of the G2/M phase cell cycle and has been a considerable predictor for early HCC formation and provides reliable biomarkers for the progression of HCC [80]. Moreover, overexpression of AURKA-induced HCC metastasis is associated with epithelial-mesenchymal transition (EMT) and cancer stem cell (CSC) behaviors controlled by the PI3K/AKT pathway [81]. The recruitment to spindle microtubules and the catalytic activity of AURKA are determined by its interaction with TPX2 [82], a microtubule-associated protein which was found to be upregulated in tumor tissues in the liver of HBV-HCC patients compared with the adjacent normal tissues in our present study. Knockdown of TPX2 inhibits cell invasion and migration as well as the tumorigenicity of HCC cells [83, 84]. KIF2C, belonging to kinesin superfamily proteins, is found to be correlated with HCC aggressiveness [85]. Of note, Kaplan-Meier analysis from our study indicated that high expression levels of TPX2 and KIF2C were associated with both the poor overall survival and relapse-free survival, suggesting that these two core genes may serve as valuable targets for both diagnosis and prognosis of HCC patients infected with HBV.

## 5. Conclusions

To sum up, the present study has identified 329 DEGs in total (67 upregulated and 262 downregulated DEGs) by integration of three profile datasets. Importantly, we obtained ten promising biomarker genes with the highest interaction degrees from the PPI network. These genes are linked to HBV-associated HCC tumorigenesis and progression and may be potential forecasters and therapeutic targets for HCC patients with HBV infection. Moreover, the multiple key biological components (e.g., extracellular exosome) and signaling pathways identified from our study will provide novel research directions concerning HBV-related HCC. Further extensive biological studies are required to corroborate our findings and to reveal the mechanisms underlying the progression of HCC induced by HBV.

## Figures and Tables

**Table 1:** 329 differentially expressed genes (DEGs) (67 upregulated and 262 downregulated) filtered by the integration of three microarray datasets.

DEGs	Gene name
Upregulated	SPINK1 TKT HSPB1 TPX2 S100P CCNB1 CLGN ASPM FLVCR1 CCDC34 AKR1B10 GINS1 KNL1 KPNA2 SMYD3 BIRC5 STIL UBE2C THBS4 FOXM1 EZH2 CCNB2 PRC1 CDK1 FABP5 CENPW RACGAP1 FANCI CENPU AURKA MCM3 DTL FAM83D TOMM40L HMMR THY1 GPC3 CDKN2C CCL20 SPP1 KIF2C SQLE LCN2 AKR1C3 SFN PEG10 UHRF1 HN1 ZWINT CDKN2A NDC80 KIAA0101 REG3A RFC4 OIP5 CDKN3 PBK TRIP13 PTTG1 STMN1 UBE2T CRNDE CENPF NUSAP1 CD109 TP53I3 DEPDC1
Downregulated	CYP26A1 BBOX1 XDH CXCL14 ACOT12 IGF1 HSD17B2 CYP39A1 EPB41L4A C1R PROZ C8A HRG ZG16 DEPDC7 MBL2 RCL1 SLCO1B3 SORL1 TRPM8 BCO2 DEFB1 TEK DACH1 TMEM45A FAM150B FOLH1B GHR CLEC1B CCL19 BHMT PON1 POU2AF1 STEAP3 SHBG ATOH8 DNASE1L3 BCHE HAO1 ID1 GPD1 FAM110C CRHBP ASS1 F9 IDO2 IGFALS SLC38A4 ACADL DBH TBXA2R CRP COLEC11 SLC39A5 SRD5A2 EGR1 ECM1 AKR7A3 SLC3A1 MS4A6A FCN2 CYP4V2 KLKB1 F11 MT1G ABCA8 SLC19A3 PGLYRP2 LINC01093 STEAP4 SLC22A1 ALDH6A1 ZFP36 MFSD2A ANO1 APOA5 HOGA1 CHST4 CFHR4 PPP1R3B RCAN1 MAN1C1 PANK1 PHGDH ARG1 PCK1 KBTBD11 SULT2A1 ADH1C DPYS CYP2C9 CYP2A7 CYP2E1 SULF2 CTH CLEC4M GABARAPL1 ESR1 ADAMTSL2 RND3 IL1RAP RDH16 ANG DMGDH TMEM27 AFM HPGD MFAP3L THRSP CYP4A11 AGXT2 NR4A3 MT1X SERPINF2 S100A8 C7 MRC1 TBX15 BMPER AADAT CCL2 NNMT SERPINA4 EPHX2 APOF GCDH FAM13A GADD45B GRAMD1C SLC7A2 TUBE1 GREM2 SDS ETNPPL DPT PRKAR2B LUM HPD SLC25A47 FLJ22763 SKAP1 EXOC3L4 SLC10A1 NAMPT ACADSB MT1E ANXA10 TTC36 GYS2 SH3YL1 ETFDH CD5L LPA C8B CXCL2 SLC22A7 TAT LIFR CYP4F2 PLGLB2 COLEC10 VNN1 LYVE1 CYP2C18 FGA FOS ALDH8A1 NAT2 MASP2 AKR1D1 PAMR1 CXCL12 GNMT TACSTD2 A1BG ACSL1 SLC16A4 CA2 FBP1 ADH4 OIT3 GLYAT ADH1A ANGPTL6 CFTR CETP INMT HBB SRPX ENO3 LECT2 ADAMTS13 PLG SOCS2 SLC13A5 CCL5 SPP2 HAO2 ACMSD IL33 HHIP ADH1B KCNN2 GSTZ1 LY6E CPS1 CNDP1 TKFC FCN3 ACACB GBA3 PDGFRA RNF125 CLEC4G OTC CDH19 FXYD1 HPX KMO ANK3 FOSB FAM65C SLC27A2 MARCO ADH6 LCAT MT1H TDO2 VIPR1 IGFBP3 PLAC8 GPT2 CFP CYP8B1 CD69 FTCD CIDEB TFPI2 LHX2 STAB2 HGFAC PTH1R MT2A NRG1 ADGRG7 CDA ZGPAT OGDHL PZP CYR61 CP HSD17B6 SLC27A5 JCHAIN GLS2 SLC51A C6 PTGIS C9 FBLN5 CDHR2 OAT

**Table 2:** The top 10 hub genes identified by the degree method of the plugin CytoHubba in Cytoscape software.

Gene symbol	Description	Degree
CDK1	Cyclin-dependent kinase 1	41
CCNB1	Cyclin B1	37
CCNB2	Cyclin B2	37
PBK	PDZ-binding kinase	34
ASPM	Abnormal spindle-like microcephaly associated	34
NDC80	Nuclear division cycle 80	33
AURKA	Aurora kinase A	33
TPX2	Targeting protein for xenopus kinesin-like protein 2	32
KIF2C	Kinesin family member 2C	32
CENPF	Centromere protein F	32

## Data Availability

The GEO database (https://www.ncbi.nlm.nih.gov/geo/) provides the microarray data to support the findings of this study.

## Abbreviations

ASPM: Abnormal spindle microtubule assembly
AURKA: Aurora kinase A
CCNB1: Cyclin B1
CCNB2: Cyclin B2
CDK1: Cyclin-dependent kinase 1
CENPF: Centromere protein F
DEGs: Differentially expressed genes
GEO: Gene Expression Omnibus
GO: Gene Ontology
HBV: Hepatitis B virus
HCC: Hepatocellular carcinoma
KEGG: Kyoto Encyclopedia of Genes and Genomes
KIF2C: Kinesin family member 2C
NDC80: Nuclear division cycle 80
PBK: PDZ-binding kinase
PPI: Protein-protein interaction network
TPX2: Targeting protein for xenopus kinesin-like protein 2.

## Supplementary Materials

Supplementary Materials Supplementary Tables Lists of GO (Table S1) and KEGG (Table S2) enrichment analysis for DEGs using DAVID.
