# Supplementary material for: Integrated Bioinformatic Analysis Identifies Networks and Promising Biomarkers for Hepatitis B Virus-Related Hepatocellular Carcinoma
Source: Int J Genomics. 2020 Jul 28;2020:2061024. doi: 10.1155/2020/2061024 (PMC7407030; doi:10.1155/2020/2061024)
Supplement: Supplementary Materials — Supplementary Tables Lists of GO (Table S1) and KEGG (Table S2) enrichment analysis for DEGs using DAVID. [file 2061024.f1.pdf]

Table S1. Summary of Gene ontology (GO) enrichment terms related to the differentially expressed genes (DEGs)

Category	Term	Description	Count	P-Value	Genes
BP	GO:0051301	Cell division	12	1.13E-04	*FAM83D, CCNB1, CDK1, CCNB2, ZWINT, TPX2, BIRC5, AURKA, PTTG1, UBE2C, ASPM*
BP	GO:0007067	Mitotic nuclear division	8	0.003429	*FAM83D, CDK1, CCNB2, ZWINT, CENPW, PTTG1, ASPM*
BP	GO:0006954	Inflammatory response	11	0.00705	*CCL2, CCL20, CRHBP, KLKB1, CXCL2, TBXA2R, CCL19, CCL5, CXCL12, ECM1*
CC	GO:0070062	Extracellular exosome	86	2.82E-13	*STEAP4, NAMPT, S100A8, MASP2, SORL1, ANO1, CRP, SPINK1, CXCL12, BBOX1, PRKAR2B, TP53I3, GPC3, TKFC, LCAT, PGLYRP2, PROZ, SERPINA4, SHBG, F11, ALDH6A1, CDK1, CDHR2, FBP1, LIFR, IGFALS, F9, CFTR, SLC3A1, THY1, C8A, C8B, RND3, CTH, TACSTD2, SERPINF2, BHMT, HAO2, HSPB1, CA2, STMN1, AKR1D1, SLC27A2, C7, ACADSB, C9, ASS1, LUM, C6, PTH1R, JCHAIN, DPYS, KMO, SFN, ARG1, ANGPTL6, KLKB1, ENO3, HRG, SLC39A5, SPP2, SPP1, DPT, THBS4, GPD1, HOGA1, EPHX2, FTCD, ACMSD, CD5L, TKT, RACGAP1, ECM1, MAN1C1, PCK1, A1BG, LYVE1, AFM, GLYAT, HPX, FBLN5,*

					PON1, CP, HPGD, IGFBP3, FABP5
CC	GO:0072562	Blood microparticle	13	7.61E-09	*C9, JCHAIN, CD5L, C8A, A1BG, AFM, BCHE, HPX, SERPINF2, APOA5, PON1, HRG, CP*
CC	GO:0005615	Extracellular space	39	2.25E-06	*XDH, MBL2, CCL2, S100A8, ADAMTS13, CRHBP, LUM, CRP, CXCL2, SORL1, CD109, SPINK1, CCL5, PTGIS, CCL20, BCHE, ANG, KLKB1, PROZ, SERPINA4, ENO3, NRG1, SPP1, THBS4, DPT, F11, IGF1, CCL19, F9, DBH, ECM1, BMPER, SERPINF2, TACSTD2, FBLN5, PON1, HSPB1, IGFBP3*
CC	GO:0005739	Mitochondrion	29	0.002339	*ETNPPL, ACADSB, OGDHL, BBOX1, ACSL1, GSTZ1, SLC25A47, DMGDH, GPT2, FLVCR1, AADAT, ALDH6A1, GCDH, GPD1, CDK1, GABARAPL1, OTC, HOGA1, ACACB, ACADL, TAT, GLYAT, ANXA10, SDS, HAO2, AGXT2, SLC27A2, OAT, BCO2*
CC	GO:0005576	Extracellular region	18	0.009137	*F11, C7, C6, PAMR1, C1R, COLEC11, PLG, LCN2, REG3A, CXCL14, PROZ, TEK, PON1, LECT2, DEFB1, TFPI2, GHR, SPP1*
MF	GO:0020037	Heme binding	14	8.69E-07	*CYP2C18, IDO2, CYP26A1, CYP2E1, CYP4V2, CYP4A11, TDO2, PTGIS,*

					CYP39A1, HRG, CYP4F2, CYP8B1, HBB
MF	GO:0008009	Chemokine activity	8	6.33E-06	*CCL2, CXCL14, CCL20, CXCL2, CCL19, CCL5, CXCL12*
MF	GO:0016491	Oxidoreductase activity	12	1.11E-05	*XDH, HAO1, AKR1C3, AKR1B10, ADH1C, ADH6, HSD17B6, CP, MCM3, RDH16, AKR1D1*
MF	GO:0005506	Iron ion binding	13	2.99E-05	*XDH, CYP4A11, CYP39A1, PTGIS, CYP2C18, CYP26A1, CYP2E1, CYP4V2, CYP4F2, CYP8B1, HBB, BBOX1*
MF	GO:0030170	Pyridoxal phosphate binding	8	1.25E-04	*ETNPPL, AADAT, CTH, SDS, AGXT2, TAT, GPT2, OAT*
MF	GO:0042802	Identical protein binding	9	0.006021	*ETNPPL, UHRF1, ASS1, BCHE, FBP1, CIDEB, AGXT2, LECT2, OAT*

Table S2. Kyoto Encyclopedia of Genes and Genomes (KEGG) pathway analysis of differentially expressed genes (DEGs).

Term	Description	Count	P-Value	Genes
bta01100	Metabolic pathways	65	2.46E-10	*ETNPPL, NAMPT, CNDP1, CYP2C18, OGDHL, ADH1C, TDO2, PTGIS, TKFC, GSTZ1, GPT2, HPD, AADAT, ALDH6A1, OTC, FBP1, CYP26A1, CYP2E1, ACADL, TAT, CTH, SDS, PANK1, SQLE, AKR1B10, BHMT, HAO2, OAT, AKR1D1, SLC27A5, XDH, ACADSB, HSD17B2, FOLH1B, ASS1, ADH6, DPYS, KMO, GLS2, ARG1, ACSL1, ADH4, ENO3, CDA, DMGDH, HSD17B6, GCDH, HOGA1, EPHX2, ACMSD, FTCD, IDO2, TKT, ACACB, DBH, CPS1,*

				MAN1C1, PCK1, GBA3, PHGDH, AGXT2, CYP4F2, CYP8B1, RDH16
bta04610	Complement & coagulation cascades	14	1.64E-08	*F11, C7, MBL2, C9, MASP2, C6, F9, C1R, PLG, C8A, C8B, FGA, SERPINF2, KLKB1*
bta01230	Biosynthesis of amino acids	11	6.36E-06	*ARG1, CTH, ASS1, SDS, OTC, PHGDH, ENO3, TKT, CPS1, TAT, GPT2*
bta04976	Bile secretion	10	3.12E-05	*SLCO1B3, SULT2A1, SLC22A7, KCNN2, CFTR, CA2, SLC51A, SLC27A5, SLC10A1, SLC22A1*
bta01130	Biosynthesis of antibiotics	17	3.18E-05	*AADAT, GCDH, ASS1, OTC, OGDHL, FBP1, TKT, TAT, PCK1, ARG1, CTH, SDS, SQLE, HAO2, PHGDH, ENO3, OAT*
bta04115	p53 signaling pathway	10	4.44E-05	*STEAP3, CCNB1, CDK1, TP53I3, CDKN2A, CCNB2, IGF1, SFN, GADD45B, IGFBP3*
bta00220	Arginine biosynthesis	6	6.63E-05	*GLS2, ARG1, ASS1, OTC, CPS1, GPT2*
bta05020	Prion diseases	7	9.42E-05	*C8A, EGR1, C8B, C7, C9, C6, CCL5*
bta00380	Tryptophan metabolism	8	9.84E-05	*AADAT, GCDH, TDO2, OGDHL, IDO2, ACMSD, KMO, INMT*
bta01200	Carbon metabolism	11	2.68E-04	*ALDH6A1, TKFC, SDS, HAO2, OGDHL, PHGDH, FBP1, ENO3, TKT, CPS1, GPT2*
bta00350	Tyrosine metabolism	7	3.40E-04	*ADH4, ADH1C, ADH6, GSTZ1, DBH, TAT, HPD*
bta00260	Glycine, serine and threonine metabolism	7	3.91E-04	*CTH, SDS, BHMT, PHGDH, DMGDH, AGXT2, GNMT*
bta00071	Fatty acid degradation	7	3.91E-04	*GCDH, ACADSB, ACSL1, ADH4, ADH1C, ADH6, ACADL*
bta03320	PPAR signaling pathway	8	0.001359	*ACSL1, APOA5, CYP8B1, ACADL, SLC27A2, SLC27A5, FABP5, PCK1*
bta00250	Alanine, aspartate and	6	0.001545	*GLS2, ASS1, FOLH1B,*

	glutamate metabolism			*AGXT2, CPS1, GPT2*
bta00830	Retinol metabolism	7	0.002098	*CYP2C18, ADH4, ADH1C, ADH6, HSD17B6, CYP26A1, RDH16*
bta04110	Cell cycle	10	0.003013	*CCNB1, CDK1, CDKN2A, CCNB2, CDKN2C, SFN, PTTG1, MCM3, GADD45B*
